# CryoEM grid preparation: a closer look at advancements and impact of preparation mode and new approaches

**DOI:** 10.1042/BST20231553

**Published:** 2024-06-12

**Authors:** Isobel J. Hirst, William J.R. Thomas, Rhiannon A. Davies, Stephen P. Muench

**Affiliations:** 1 School of Biomedical Sciences, Faculty of Biological Sciences, University of Leeds, Leeds LS2 9JT, U.K.; 2 Astbury Centre for Structural Molecular Biology, University of Leeds, Leeds LS2 9JT, U.K.

**Keywords:** cryo-electron microscopy, sample preparation, single particle cryoEM

## Abstract

Sample preparation can present a significant hurdle within single particle cryo-electron microscopy (cryoEM), resulting in issues with reproducibility, data quality or an inability to visualise the sample. There are several factors which can influence this, including sample or buffer composition, grid type, route of sample preparation and interactions with the air–water interface (AWI). Here, we review some of the current routes for sample preparation and the associated challenges. We discuss a range of approaches for overcoming these challenges, such as minimising the grid preparation time, surfactants, grid type and biochemical approaches such as nanomagnetic beads. Finally, we discuss how a set of commercially available protein samples may serve as a benchmark suite for future technologies. This provides a route to compare techniques’ abilities not just to generate high-resolution structures but also to overcome the challenges traditionally associated with cryoEM. As the field continues to produce new approaches to sample preparation and we start to better understand the underlying principles behind the behaviour of proteins within a thin film and in response to different environments, especially grid composition, it is hoped that more universal solutions can be provided that make the intractable systems tractable, improve resolution and, importantly, speed up data collection and reduce the currently required dataset sizes.

## Introduction

Single particle cryo-electron microscopy (cryoEM) has become a go-to technique within structural biology due to its amenability to samples that, with other techniques, are challenging targets. For example, many classes of membrane proteins have now become more routine in their structure determination including GPCRs, TRP channels and large complexes that had typically been hard to structurally determine [1]. With advances in microscope and detector hardware [2] and constantly improving data processing routines, the breadth of samples that can be studied, and the resultant resolution is improving year on year [3]. However, although there have been many success stories within the field, sample preparation is still a bottleneck and is comparable to the crystallisation phase in X-ray crystallography, with overall success or failure often depending on its outcome [4]. Moreover, a significant proportion of a typical cryoEM dataset is discarded during image processing to remove ‘badly behaved' particles. These can include contamination, degraded protein and inconsistent ice quality. Poor particle distribution within the ice and/or preferred orientation consequently typically requires a ‘brute force' approach where significantly more micrographs are collected to compensate for the poor quality data these problems produce. Collection of larger datasets decreases throughput not only due to the increased amount of microscope time required, but also the larger amount of time it takes to process large datasets, not to mention the strain it puts on the provision of data storage. Therefore, reduced sample damage and a higher level of control during grid preparation will not only increase the proportion of good-quality particles in a collected dataset but will increase the overall efficiency of cryoEM by reducing the number of grids and grid conditions that need to be screened to produce a collectable grid.

Typically, an ideal grid will have ice ∼20 nm thicker than the specimen to avoid too much variation in defocus between particles or overlapping layers of proteins. Within the ice, the particles should be intact, monodisperse, display a range of views, and show no signs of denaturation or other modifications [5]. A myriad of aspects of the grid preparation process mean that this ideal is rarely reached. Here, we review the most pressing causes of challenges in grid preparation and some of the established and emerging approaches for overcoming them. We do not cover the basic principles of sample preparation and refer the readers to the following papers which give an excellent introduction to the field [4,6].

## Consistency of ice quality

One approach to sample preparation advancement is through the development of freezing devices with increased automation, improving control of freezing conditions and therefore consistency. Currently, the most common freezing devices, the Vitrobot and Leica EM GP systems (hereon referred to as ‘standard blotting devices'), also have the most manual steps. Grid preparation can be split into four main steps: (1) application of sample onto the grid, (2) thinning of the liquid, to produce a thin film (3) vitrification to produce a thin vitreous ice layer and (4) storage of the grid in the grid box. Of these four steps, only steps 2 and 3 are done automatically in standard blotting devices, with the user required to complete the rest of the process manually, a technical process that takes time to master [7].

Whilst manual processes inevitably result in greater inconsistency from grid to grid, another major source of inconsistency with standard blotting devices is the method of thinning the sample layer on the grid through blotting with filter paper. Two grids can often be made using these devices with the same sample, blot force and time, filter paper type, and by the same operator and yet both can have different ice qualities, although it should be noted that consistency can be improved through correct setup and maintenance [8]. Despite these inconsistencies, the use of this method by no means precludes the determination of high-resolution structures. Indeed, the vast majority of high-quality structures in the Electron Microscopy Databank (EMDB) are from grids prepared using standard blotting devices [9]. The consistent production of grids with desirable ice would diminish the time-consuming need to make and screen an excess of grids as well as open the door for grid multiplexing, increasing throughput in multiple ways. To this end, various devices have been developed over the past decade that seek to improve the reproducibility of ice on grids through increased automation and alternative approaches to blotting.

Some of the devices that have been developed to address the challenge of reproducible vitrification of grids, and the ways they address this, are listed in Table 1. A common approach is to deposit a considerably smaller volume of sample onto the grid to reduce, or in the case of the VitroJet completely eradicate, the need for sample thinning on the grid [10]. Both the chameleon [11] and the cryoWriter [12] apply a volume of only a few nanolitres so that a thin liquid film can be formed from thinning by either self-wicking grids in the case of the chameleon or controlled evaporation in the case of the cryoWriter. The CryoGenium [13] takes a completely different approach, where the grid is dipped into the sample and the excess is sucked off. One thing that all these devices have in common, and that is fundamental to the delivery of more reproducible ice thickness, is the ability to monitor sample thinning in real-time and provide a relative ice thickness estimate. This allows the settings to be adjusted to produce desirable ice thickness for a sample within a single freezing session and provides assurance that the sample thickness will be within the ballpark of the target thickness before screening in a cryo-electron microscope. This is a significant step forward for increasing the throughput of cryoEM and although development is still ongoing and not yet mature, on-the-fly assessment of ice quality will be a significant advantage moving forward. Microscope time is expensive and often has lead times of weeks or months so it is important to ensure that all the grids are of sufficient quality. The monitoring of sample thickness during the freezing session and the fine control over the extent of sample thinning enables optimal conditions to be determined on a per-sample basis. Consequently, once the optimal parameters have been determined, grids with suitable ice thickness can be reliably reproduced.

**Table 1.** Overview of some of the available vitrification devices and their approaches to improve ice thickness and reproducibility

Device name	Sample deposition	Thinning of sample on grid	Vitrification	Storage of grid	Time between deposition and vitrification	Inline glow discharge?
Vitrobot	Manually with a pipette	Double-sided blotting with filter paper	Plunge into liquid ethane	Manual	>5 s	No
Leica EM GP(2)	Manually with a pipette	One- or sequential two-sided blotting with filter paper	Plunge into liquid ethane	Manual	>5 s	No
chameleon	Inkjet piezo dispenser, nl of sample in a stripe on a grid	Self-wicking nanowire grids	Plunge into liquid ethane	Automatic	54 ms–2 s (user chooses)	Yes
VitroJet	Contact pin printing of sub-nl volume	Not required	‘Jet vitrification’ — liquid ethane onto pre-clipped grid	Automatic	Seconds	Yes
cryoWriter	Microcapillary writing of 1–2 nl of sample	Evaporation (but humidity controlled and has a ‘climate jet’ and ‘coverslip injector’ to protect from AWI interactions)	Plunge into liquid ethane	Automatic	<1 to 10 s	Yes
CryoGenium	Dip grid in sample	Sucked away with tubes	Plunge in liquid ethane	Automatic	30–60 s	Yes

Another significant feature that these devices have in common is the reduction in manual handling steps. In the case of the cryoWriter, there are no manual handling steps as the grid is removed from the storage box automatically and the VitroJet, chameleon and CryoGenium require the user to manually load the grids into the device, but all subsequent handling steps are automatic. As grids are small, delicate and require cryogenic storage temperatures, reduction in manual handling reduces the likelihood of foil breakage, deformation and devitrification. This improves the reliability of the production of good-quality samples and the likelihood of the grids that make it into the microscope being usable. It also makes grid preparation less technical and therefore more accessible, increasing the range of scientists that can easily incorporate it into their research and therefore broadening the applicability of cryoEM as a technique. Even for experienced users, increased automation may increase the success rate of prepared grids and reduce sample wastage which is another step towards cryoEM as a high-throughput technique, as would be required for its use in structure-based drug design, for example. Another advantage of automation is that the conditions used on a given device can be accurately reproduced by other users. This opens up the possibility for a database where grid preparation parameters used for a given sample as well as the quality of the resulting grids can be deposited, allowing the community to build on each other's work by enabling the sharing and reliable replication of optimised protocols for more difficult samples.

Finally, the setup of the cryoWriter, chameleon and VitroJet all involve controlled application to specific areas of the grid, opening up the opportunity for the application of multiple different samples to different areas of the same grid (multiplexing) as well as the addition of one sample on top of the other in the same part of the grid which could permit time-resolved cryoEM.

## Problems beyond ice thickness and consistency

**Figure 1.**
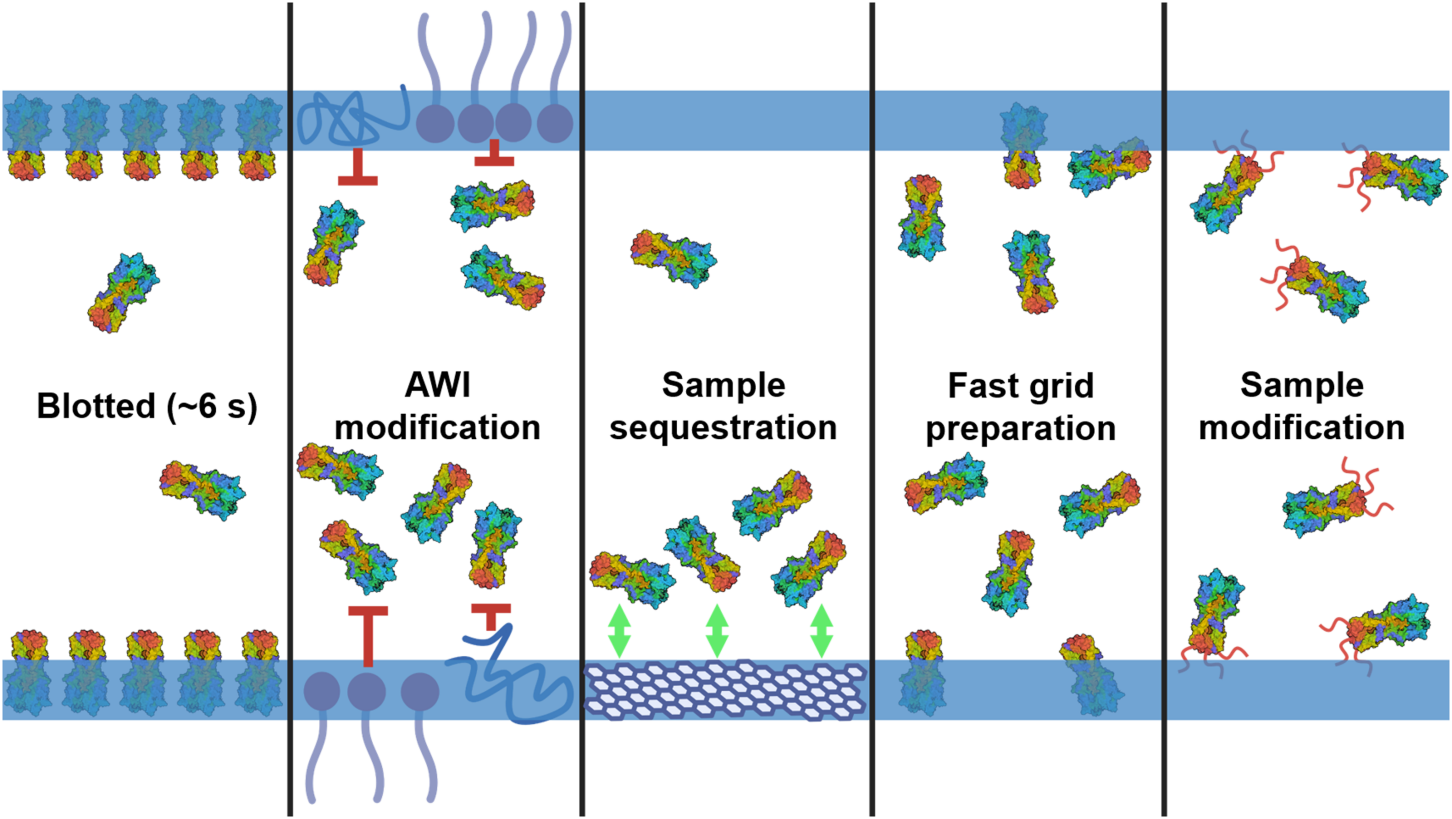
Summary of the approaches taken to mitigate cryoEM sample preparation issues. The protein sample (HA trimer, PDB 7VDF) is shown within the liquid thin film. The film's boundaries, representing the AWI, are denoted by two horizontal blue lines. The panels (left to right) represent adsorption to the AWI during the blotting process, modifying the AWI to reduce interactions with the AWI, sequestering the sample away from the AWI through a support film, reducing the interactions through faster sample preparation on millisecond timescales and modifying the sample to change its properties and interactions with the AWI. This figure was created with BioRender.com.

Although optimal ice thickness permits high-resolution data collection it does not guarantee it. An ideal cryoEM grid has a single layer of monodisperse particles in a range of orientations within the thin ice layer. The requirement for thin ice results in a thin liquid film of sample being formed on the grids between sample application and vitrification and here lies the other major source of difficulties in sample preparation for cryoEM (Figure 1). A thin liquid film presents a unique chemical and physical environment which can have detrimental effects on protein samples. There are numerous ways in which sample behaviour on cryoEM grids can be suboptimal as well as many adaptations that can be made in response to the challenges on a sample-by-sample basis.

The source of the majority of difficulties in grid preparation for cryoEM is the air–water interface (AWI) [14,15]. The thin film of the sample on the grid prior to vitrification has a high surface area to volume ratio so the sample is exposed to an extensive AWI on one or both sides of the thin liquid film depending on the type of support film used. The various deleterious effects of protein interactions with AWIs have been well researched in other contexts as well as increasingly in the context of grid preparation for cryoEM [16]. The main issues caused by the environment of the thin liquid film and the most recent approaches to mitigate them are outlined in the following sections.

## Preferred orientation

A common problem in cryoEM is that the protein sample adopts a preferred orientation within the thin film often as a result of AWI interactions. The resulting limited range of views can cause significant problems in structure determination with map anisotropy, stretching and smearing of density. A common way to overcome this problem is to modulate the way the protein interacts with the AWI using surfactants such as detergents [17]. For example, FOM was used to change the orientational preference of a SARS-Cov-2 antibody complex [15] and fos-choline-8 has been shown to influence the ClC-1 chloride channel [19]. DDM, CHAPS and CHAPSO have also successfully been used for other soluble proteins including aldolase, β-galactosidase [20] and human erythrocyte catalase [21]. One limitation of this approach is that the rules determining the most appropriate detergent are poorly understood and therefore a trial-and-error approach is required, not just for the type of detergent but also the sample concentration required. Moreover, a higher sample concentration may be required. This may not be viable for some samples due to their tendency to precipitate and/or inability to express/purify sufficient quantities of the sample [21].

An alternative approach to overcoming preferred orientation is to limit the effects of interactions with the AWI through speed. Many studies have shown that although particles localise to the AWI too quickly for AWI interactions to be completely out-run [22], devices that make grids rapidly (typically <200 ms) result in improvements in angular distribution as seen with the cryoWriter [12], chameleon [15] and homemade spray devices [23,24].

Preferred orientation can alternatively be addressed during data collection by tilting the specimen stage to increase the range of views in the micrographs. However, the increased ice thickness in micrographs collected at a tilt as well as the differences in defocus make this a less desirable solution than the avoidance of the preferred orientation in the first place [25].

In contrast with the environment being modified, the sample itself may be modified to confer favourable characteristics. For example, primary amine PEGylation has been shown to protect proteins such as NOD2 from aggregation and denaturation [26]. Even relatively ubiquitous protein modifications such as His-tagging have the potential to alter interactions with the AWI [27]. Alternatively, the sample can be associated with a protein scaffold. This can enable certain conformations to be stabilised [28], the angular distribution to be improved [29] and the structure of small proteins to be solved [30]. However, these benefits are contingent on the binding modality and the scaffold's geometry/symmetry.

## Ideal particle concentration and distribution

Another problem can be the sample preferentially localising on the sample support film, leading to a low observed protein concentration within the ice. This can be due to the thin liquid film not accommodating the size of the particle, thereby excluding it to the edge of the hole where the liquid layer is thicker or requiring imaging in areas of thicker ice. A common approach to alter particle distribution and concentration is to use variations in hole size and/or grid geometry. This can be particularly effective for particles that tend to accumulate in specific ice thicknesses with smaller holes typically giving thinner ice and larger holes and larger mesh sizes a ‘flatter' thickness gradient to help increase the usable area. The smaller film area may help to force particles into the hole rather than accumulate on the support film. The cause of the protein sample preferentially occupying the support film rather than the holes may also be due to interactions between the support film and the protein sample itself, in which case modification of the support film is required to make the support less attractive to the protein. This can be achieved by glow discharge in alternative plasmas such as those made from amylamine or methanol which coat the support in positively or negatively charged hydrocarbons, respectively [31]. Although holey carbon is the most commonly used type of support film, with samples that have an affinity for the carbon in the holey carbon film, support films made of other materials such as gold often alleviate the problem and allow the sample to populate the holes. Alternatively, a continuous, ultra-thin substrate can be added on top of the holey support film, with popular choices including graphene, graphene oxide or amorphous carbon. Sample adsorption to this continuous film helps to concentrate the protein in the holes whilst simultaneously sequestering particles away from the AWI. Moreover, the surface of these continuous support films can be modified to increase the affinity for the sample such as the use of streptavidin crystals to isolate biotinylated samples [32], epoxidised graphene grids [33] or PEGylated gold grids [34]. Affinity grids to isolate specific tags such as His tags have long been sought after and new approaches are starting to be developed, including the use of affinity tags for on-grid protein purification from cell lysate [35].

Whilst a single continuous support film generally has the desired effect of minimising AWI interactions by secluding particles away from it (although this can result in preferred orientations and/or support film-sample interactions), there has long been interest in the concept of a ‘coverslip’ for cryoEM which is generally used to refer to something that covers both sides of the sample, removing any AWIs from the sample support set up. The cryoWriter offers one approach to this in the form of its ‘coverslip injector’ which allows the user to coat the AWI on either side of the thin film with effector molecules of their choice, enabling the use of a much wider range of molecules as it never needs to be added directly to the protein sample itself. Another approach to the concept of a coverslip is to sandwich the sample between two thin layers of a low-contrast substance. One of these approaches is the CryoChip, a sample support which sandwiches picolitres of sample between two sheets of silicon-rich nitride [36]. Another approach is the use of two layers of graphene [37]. A graphene continuous support film is added to a grid, then the sample is used to float a second layer of graphene onto the grid which is then placed on filter paper to remove excess sample. This results in an average ice thickness of 60 nm and an observed increase in particle concentration compared with the bulk solution. Sandwiching the sample in graphene has the benefit of covering both AWIs as well as reducing charging effects and particle motion due to the conductivity of graphene. Despite these advantages, the challenges involved in making, storing and handling graphene mean this is not yet a widely accessible approach.

Alternatively, the foil can be saturated with the sample by applying the sample 2 or 3 times (blotting after each application), so that an additional application of protein will enter the thin film layer across the holes in the support. A brute force approach could also be used where simply adding an excess of protein when blotting results in a sufficient amount residing within the thin film but requires a sample that can be expressed at high levels whilst remaining well-behaved at high concentrations. An interesting new approach to overcome concentration issues is the use of magnetic concentration and isolation cryoEM (MagIC) [38]. This technique binds GFP-labelled proteins via spacer constructs ending in biotin to streptavidin-coated nanomagnetic beads. These MagIC beads enable the concentration of the protein sample in the holes when the grid is held above a magnet, allowing the use of samples at 100- to 1000-fold lower concentrations than typically used for cryoEM [38]. Another route to overcoming limited sample availability is through the cryoWriter which can purify and dispense a protein within the sample deposition nozzle allowing small cell volumes to be used to generate high resolution single particle cryoEM structures [39].

## Protein modifications (oxidation, denaturation, subunit dissociation)

Even when the sample is well distributed in the thin film with a good angular distribution, many problems can persist. There have been several studies looking at the change in the chemical environment when going from a bulk solution to a thin film which includes a highly charged surface, the production of damaging chemicals such as peroxide [40] and in the case of blotting there can be leaching from the filter paper of contaminants such as divalent metals [41]. Although outside the scope of this review, there is an excellent review on the chemistry of thin films and droplets and the ability to catalyse reactions here [40]. These all combine to create problems with the protein itself. For example, Kühlbrandt and co-workers [16] showed denaturation of the PPT domain of the fatty acid synthase complex was propagated at the AWI. Other work has shown that different subunits of the ribosome can be lost during sample preparation in a time-dependent manner, indicative of protein degradation/dissociation over time [24]. Moreover, the chemical modification of cysteine residues has also been noted to occur in a time-dependent manner within the thin film environment [42].

## Future directions

The grid preparation field has grown significantly over the last few years, especially in the variety of approaches. However, without a set of ‘standard' proteins it can become difficult to gauge the most appropriate approach for different problems with resolution often being the sole discriminator. We propose a standard set of proteins should be employed across studies focused on grid preparation methodology. Such proteins would allow the cryoEM community to more easily benchmark proposed improvements in cryoEM grid preparation methodologies, offering greater insight into how broad their application is likely to be (Table 2). The haemagglutinin trimer exhibits a strong preferred orientation and has accordingly already become somewhat of a standard in many studies to demonstrate the effectiveness of methodologies tackling this problem. Comparisons between methodologies have been aided by the development of quantitative metrics that indicate the extent of preferred orientation bias by considering Fourier space sampling [43] and directional resolution [44]. Additionally, the plunge time-dependent loss of the L31 and S2 subunit from the ribosome on a millisecond and second timescale, respectively, shows its suitability as a benchmark sample for subunit dissociation on the millisecond timescale [24].

**Table 2.** **Overview of proposed standard proteins to facilitate** **comparison of new developments in sample preparation for cryoEM**

Proposed model protein	Issue that it represents	Example catalogue number
HA trimer	Preferred orientation: majority of particles exhibit ‘top’ views [44]	Bio-Techne **10973-HA-100**
70S ribosome	Subunit dissociation: L31 disappears from density maps in a time-dependent manner [24]	New England Biolabs *E. coli* 70S ribosome **50-443-9**
Alcohol dehydrogenase	Low contrast resulting in challenges in particle alignment: 82 kDa protein with low order of symmetry (C2) [45]	Sigma–Aldrich equine ADH **55689**
β-Galactosidase	Cysteine modification [42]	Sigma–Aldrich *E. coli* β-galactosidase **G3153**. Purity has been reported to be batch-dependent, so an SEC cleanup step is required.

Another protein sample characteristic that can introduce challenges is size with smaller samples <150 kDa, having lower contrast in micrographs and being more difficult to accurately align during image processing. Therefore, it is important to demonstrate the benefits of sample preparation approaches on both larger and smaller samples to show the translation of the method with smaller samples, especially those with lower orders of symmetry. Here we propose the use of horse liver alcohol dehydrogenase, an 82 kDa protein with C2 symmetry, to give an impression of the amenability of new sample preparation approaches to smaller proteins. β-Galactosidase also provides a good test for Cys modifications which have been shown to occur in a time-dependent manner [42]. Importantly, all of these samples are commercially available, facilitating their incorporation into a method validation workflow. It should be noted that the protein quality can vary between batches, especially for β-galactosidase. Therefore, it is recommended to do protein purification and quality control (gel analysis and/or negative stain analysis) before sample preparation.

## Perspectives

- There have been significant developments in the field of grid preparation and although challenges remain we are now in a position to better understand and control some of the negative factors present.
- With so many new approaches we can now start to understand how factors such as preparation time, grid type, surfactants, the AWI and buffer compositions can influence the resultant grid and devise new ways of tackling these. By working on a common set of proteins we can start to compare approaches based on factors other than resolution and broaden our toolkit for grid preparation.
- Looking forward, as data collection speeds improve but grid loading and initial analysis remain constant, an important way to improve throughput will be to circumnavigate these slower steps through multiplexing on grids and improving the ratio of good to bad particles within a dataset.

## Open Access

Open access for this article was enabled by the participation of the University of Leeds in an all-inclusive *Read & Publish* agreement with Portland Press and the Biochemical Society under a transformative agreement with JISC.

## Abbreviations

AWI: air–water interface
cryoEM: cryo-electron microscopy
MagIC: magnetic concentration and isolation cryoEM
